# Curing Consumption

**Published:** 1859-08

**Authors:** 


### CURING CONSUMPTION.

Pure air contains one part of oxygen and four parts of nitrogen, and the habitual breathing of an impure atmosphere, one which has not its due supply of oxygen, is a very common cause of consumption of the lungs. But while the want of oxygen in the air originates consumption, it is quite as true, that an atmosphere which has one fourth less oxygen, is curative of consumption, for it is found that mountaineers, living at an elevation of twelve thousand feet, are almost exempt from this disease; such an elevation involving a less amount of oxygen in a given amount of air, by one fourth, than is found at the ordinary level.

That "consumption decidedly diminishes in elevated situations," is an indisputable fact; so that certain physical conditions may cause consumption, and also may cure it. Yet our announcement several years ago, that whatever might be the effects of "tight lacing" in causing consumption, it had a tento arrest and cure it, made some merry, and other some mad. How such a statement should have had such opposite effects on persons who were equally daft, we shall not stop to inquire. But the *rationale* of a rarified atmosphere and of tight lacing as a means of arresting and curing consumption, is the same, and is beautifully philosophical. They both cause quicker breathing, "more labored breathing, producing an increased expansion of the lung, agreeing perfectly with the fact, that the summit of the lung is principally, or almost solely the seat of tubercles, and with the probability, that is the result of the less degree of dilatation, which precisely this part of the lungs must experience in consequcnce of the conical shape of the chest."

Now this quotation, which is worthy of being written in letters of diamonds bright, is taken from the pages of Messrs. Wood's re-print of the British and Foreign Medico-Chirurgical Review, No. 45, the soundest and ablest medical periodical in the English language, within our knowledge.

It means simply this, that tubercles are found almost exclusively at the top of the lungs, immediately under the collar bones, and that is the spot where consumption begins to cause the lungs to decay, in almost every instance.

The reason that tubercles are prone to form there is, the bony case which surrounds the lungs there, prevents the full expansion of the air cells; but lower down, near the edges of the ribs, tubercles are almost never found, even in the last stages of consumption, because the ribs there, are distensible at each inspiration, and that enables the air cells, the lungs themselves, to distend to their utmost, habitually, which habitual distension is utterly incompatible with the existence of tubercle and consequent consumption.

Hence, the mountaineers, by their quicker and "more labored breathing," attain a structure of chest so broad at the top, as to be remarkable, and their lungs are so highly developed, as to present an extraordinary appearance.

A stranger once came to us to ascertain our manner of treating consumption, as he thought he had cured himself of it, and was curious to know if his principles and ours were similar. They were, and both involved this "labored breathing," by artificial means.

In the second edition of our dollar work on consumption, just issued, these things are referred to at length. Go then to the cold, rarified mountain-air to cure you of consumption, and not to the hot Savannahs of the south, where every breath you take is loaded with steaming moisture and disease engendering miasm, oppressing the system, taking away the strength, and corrupting the blood at every inspiration.

